# Application of a Random Forest Algorithm in Natural Landscape Animation Design

**DOI:** 10.1155/2022/2820558

**Published:** 2022-05-25

**Authors:** Licheng Zhao, Kaixin Zhang

**Affiliations:** ^1^Teachers and Design Institute, Harbin Vocational College of Science and Technology, Harbin 150300, China; ^2^Harbin University of Science of Technology, Harbin 150000, China

## Abstract

Natural landscape simulation is one of the most popular research contents in computer graphics in the field of research simulation system. The natural landscape animation scene can immerse viewers in the scene, and it is widely used in visual simulation systems. Simulating natural scenery on a computer is a powerful method for studying the rules of the scenery's growth process as well as the mystery of life. The simulation of natural scenery is of particular importance and has far-reaching implications. The most important aspect of optimizing natural landscape design is creating a natural landscape animation that users enjoy. This article proposes a natural landscape animation design method with a self-learning function based on this concept. The random forest model (RF) is introduced in this method and applied to the entire animation design process. RF can generate a learning model with user evaluation as the classification result to guide the automatic design of natural landscape animation, resulting in user-satisfying animations. Simultaneously, the RF-based natural landscape animation design can continuously update the learning model based on user needs and is self-learning. The experimental part of this article verifies the effectiveness of the natural landscape animation design proposed in this article by comparing the selection rate of user satisfaction and dissatisfaction scenes, and further demonstrates that the method in this article can improve the natural landscape. The market application value of user satisfaction generated by animation is high.

## 1. Introduction

Scene design is an essential component in the creation of animated works. The scene's design can emphasize the relationship between time and space, making the animation plot more appealing. It is still a very complicated process in the specific scene design. There must be an awareness of overall scene design, a specific cultural connotation, and attention to the processing of detailed scenes. In animation, the shaping of natural landscapes is crucial in a variety of scenes. Because nature is so inventive, shaping and processing natural landscapes is even more difficult. Now, there are three major types of research being conducted in the field of natural landscape animation design. One is that natural scenes are only mentioned infrequently in scene design literature. Most discussions about natural scenes are straightforward. A lot of research and practical application are out of sync, and theory and practice cannot be well connected. The second is an explanation in the form of a tutorial, complete with examples. Model creation, UV unwrapping, texture drawing, material adjustment, lighting settings, rendering, and postproduction special effects application are all covered in detail. This tutorial-style description covers a wide range of knowledge points, but it is insufficiently detailed. The third type of document is a single software instruction manual. This document primarily describes how to use the software. In fact, the design and production of 3D scenes in the process of animation design and production is more than just the use of a specific software. To complete the task, a combination of software is required. There have been a few studies on how different softwares are combined to complete tasks. The existing research literature does not systematically summarize the technical difficulties and key points in the actual design and production of natural scenes, and thus cannot meet the needs of natural scene design and production in high-end animation films.

Natural scene animation is primarily concerned with learning how to use a computer to generate animation of natural scenes such as white clouds, creeks, pine trees, and grass. Natural scenes are generally difficult to describe using traditional geometric modeling tools, necessitating the use of specialized modeling techniques. There is still no unified method for animating natural landscapes. People are increasingly interested in mathematical models that can accurately describe various phenomena and landscapes in the objective world as computer graphics technology advances. Dynamic natural scenes, on the other hand, are difficult to simulate with simple processes and must be reflected according to physical laws of the real world. Scholars have been attempting to investigate the method of natural scene animation for a long time, including the simulation of the natural scene's shape and the simulation of the natural scene's dynamic change process. Many scholars introduced random process theory and proposed a series of effective models to generate various specific natural scenes, considering the randomness and similarity of the shape details of natural scenes.

Most widely utilized at present are algorithms based on fractal iteration, grammar-based models, dynamic random growth, texturing, and atmospheric optical transmission models as well as algorithms based on unique geometric instances and methods utilizing interaction between the scene and the model. Modeling of plant growth on a computer screen is known as “plant modeling technology.” An early kind of plant modeling was developed in the 1960s using the automatic growth model of cells to make animations of plant branching events. Lindenmayer, an American biologist, initially introduced the concept of a string rewriting system in 1968 [1]. Later, in honor of the biologist, it was dubbed the L-system. The system is primarily concerned with the relationship between the genus's plant organs, which include the trunk, branches, and leaves. Prusinkjewicz, a Canadian academic, developed the geometric description based on the L-system, thereby establishing the open L-system [2]. The system includes communication modules that enable plants to communicate with their environment. The system can animate the root system of plants and simulate the effect of the external environment on plant growth, which is beneficial for the development of virtual agricultural production systems. Because each rule in the traditional L-system is completely independent, it cannot faithfully reflect the growth process of plants and thus cannot produce a smooth animation effect. As a result, the traditional L-system is inadequate for some applications, so Praniewicz et al. proposed the differential L-system at the World Graphics Congress in 1993 [3]. The main idea behind this system is to use a unified formal tool to describe the discrete and continuous behavior of organs. In 1995, American scholar Jason Weber proposed a modeling method that only considers the geometric shape of the tree's appearance [4], and this modeling method does not strictly adhere to the physiological structure of the tree. Other than some basic geometric knowledge, this model does not necessitate a strong mathematical or biological foundation. Greece [5] proposed a method based on voxel space. The concept of voxel space is to divide a three-dimensional space area into several cubes, with each small cube representing a volume element. Models develop in voxel space based on their intersection, neighbor, and collision relationships in element space. Volume elements are used to approximate both the plants and the surrounding environment in the space. DeReffye et al. proposed a “reference axis technology” plant modeling method [6], which describes plant morphology using initial automation and plant growth models using the Markov chain theory of stochastic processes and state transition graph. Currently, many plant animation methods, such as the particle system method stochastic model proposed by Reeves et al. [7, 8], the branch matrix model proposed by Viennot, and the interactive plant construction model based on functional icons proposed by Lintermann et al. [9], are like the L-system. The abovementioned plant animation models are primarily based on plant shape, which does not accurately reflect plant physiological models. Most of them concentrate on computer graphics, primarily studying the animation of realistic plant graphics, and employ as few plants as possible. Learn new things and create plant graphics quickly and easily.

Machine learning technology [10, 11] has become very common and mature as a result of the development and application of artificial intelligence technology. The fundamental idea behind machine learning is to construct a training model by learning the features and outcomes of known data sets, referred to as training samples. On unknown data, the trained model can make predictions. Decision trees, neural networks, support vector machines, random forests, and other traditional machine learning algorithms are examples. This article proposes applying the RF model [12, 13] to natural landscape animation design in order to optimize the effect of natural landscape design. This article's main contribution is as follows:

1. RF is used to train a learning model with user evaluation as the classification result in order to guide the automatic design of natural landscape animations that satisfy users.
2. The central idea behind applying the RF model to natural landscape animation design is to mine the experience that can guide the animation design from the accumulated animation data. RF models' abstract properties and categories A training sample set is obtained after standardizing a large amount of historical data, and an RF model is obtained after training.
3. Experiments are used to continuously optimize the RF model's parameters so that the learning model can better guide animation design and create animations that satisfy users.

## 2. Knowledge of Natural Landscape Animation Design

### 2.1. Problems Existing in Natural Landscape Animation Design

At present, the problems existing in the process of natural landscape animation design can be summarized in the following aspects.

1. Practitioners and small production companies are inexperienced and of poor professional quality. In recent years, China's animation industry has grown rapidly. However, some issues, such as a lack of professional talent, cannot be avoided in the process of rapid development. The quality of domestic 3D animation products is also low due to a lack of professional talent and low-end production. The resulting product frequently bears a distinct imitation imprint. The entire link demonstrates that the foundation is weak, from the lack of script innovation to the unsatisfactory animation generation effect. The talent structure is young, as is the 3D animation company, and the industry lacks operational experience. Furthermore, investors and planners are frequently profit-driven laypeople. As a result of these factors, there are few high-quality animations produced.
2. The mode of training for high-quality professional talents deviates from the path. The development of 3D animation industry is indeed uneven, with most of the activity concentrated in a few large cities such as Beijing, Shanghai, Shenzhen, and Chengdu, while small- and medium-sized cities are largely unaffected. An industrial layout like this has resulted in a large influx of practitioners into cities like Beijing and Shanghai. However, this has not changed the current situation of a shortage of high-quality talent, as reported by animation companies. Despite the large number of employees, there are not many high-level employees. The following aspects primarily reflect the reasons. The first is that training institutions provide instruction without enough teachers and resources. As a result, low-level production personnel and high-level pure researchers are trained, resulting in an unbalanced talent structure. Second, the training institutions themselves lack practical experience, resulting in true quality education being empty talk. Third, prioritize technology over basic training, such as modeling, composition, color, lens, and animation principles. Because basic training is time-consuming, trainees do not want to waste their time and energy. This type of operation results in students' weak foundations, a lack of solid technology, a lack of practical ability, and an inability to improve the level.
3. The script creation orientation is low, and the script content lacks innovation. There are also numerous issues with the script's creation and selection. Because of the screenwriter's misunderstanding of the concept of animation and the narrow positioning of the audience's age group, the script was created to be simple and young. Most adults have given up on cartoons due to their narrow positioning. The changing of the seasons is bound to change the viewing preferences of the audience. As a result, traditional cartoons have been rendered obsolete by the passage of time. 3D animation is a type of animation that caters to modern audiences' needs. The three-dimensional animation industry is in its prime right now, and works are increasingly focusing on both technology and plot.
4. A lack of distinguishing features and local brands. There are numerous classic stories in Chinese traditional culture, which is extensive and profound. While some stories have already been used, there is still room for improvement in their presentation. Some cartoons produced by Disney in the United States and DreamWorks in the United States in recent years are typical scripts with Chinese characteristics, and they have boldly adapted and processed the original content and prototype to make the storyline more suitable for modern audiences. Aesthetics and taste foreign animation companies not only created high-quality scripts, but also distinct animation stars and established a good animation brand in large-scale productions. Chinese cartoons, in terms of script writing and selection, blindly repeat and imitate, fail to make good use of the rich cultural history, and fail to innovate. These circumstances have stifled the growth of the 3D animation industry. Only by creating your own brand will you be able to gain a foothold in the animation industry.

### 2.2. Software for Natural Landscape Animation Design

The basic software for creating three-dimensional natural landscapes is Maya and Max [14, 15]. It is also the final carrier software to produce three-dimensional natural landscapes. Other softwares are developed to supplement these comprehensive softwares. MAYA and MAX each have unique strengths when it comes to creating three-dimensional natural landscapes. Maya and Max are frequently used in the creation of realistic natural scenes. In general, it is possible to select which software to use based on various project requirements or the producer's own expertise. These two softwares must be mastered by practitioners because they are the primary carriers of many 3D animation design software. The two softwares are becoming increasingly similar in terms of operation interface and functions, but they still have distinct characteristics. The Vue series products offer a variety of solutions for animation production and 3D natural environment rendering, making it possible to create large scenes quickly.

## 3. Natural Landscape Animation Design Based on Random Forest Model

### 3.1. Natural Landscape Animation Generation

**Figure 1:**
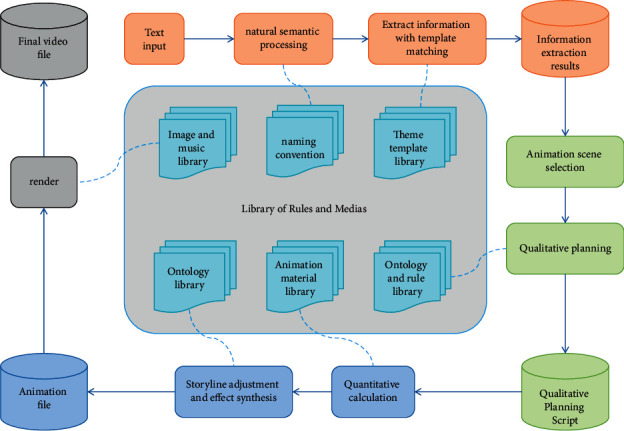
Flowchart of natural landscape animation generation.

Figure 1 depicts the flow of natural landscape animation generation. The information extraction module extracts several themes and templates from the message text after receiving it. The theme is the extraction of animatable animation theme series from the message, such as towering trees and chrysanthemums in the mountains and plains. Wind templates, sunshine templates, and so on are examples of concrete objects. For example, from the message “sunny spring, the peach blossom branches in the mountains laugh,” the action templates of the themes “spring,” “mountain,” “peach blossom,” and “smile” are extracted and stored in the IE file in turn. Verify the plot. The plot determination module uses the information framework to call the corresponding ontology library and rule library, and then uses reasoning to complete the selection of scenes, model additions and deletions, and determination of actions, colors, deformations, and lighting, among other things. The quantitative planning module performs quantitative calculations based on the qualitative planning and generates playable animation video files via network rendering. A landscape animation is created at this point.

### 3.2. Self-Learning Method of Natural Landscape Animation Based on Random Forest

The RF algorithm is a type of Bagging algorithm [16, 17]. In comparison with Bagging, RF only creates its own regulations and designs for a few details. RF refers to the bagging method that employs a CART decision tree as a weak learner. First, as a weak learner, RF used a CART decision tree [18, 19]. Simultaneously, when each tree is generated, the features selected by each tree are only a few randomly selected features, and the root of the total number of features is generally taken by default. All the features will be selected for modeling by the general CART tree. As a result, not only are the features random, but the randomness of the features is guaranteed. In comparison with the general Bagging algorithm, RF will choose N samples from the collection and training set. Because of the randomness, it is very useful to reduce the variance of the model, so the RF does not generally require additional pruning, achieving better generalization and anti-overfitting ability. Of course, the degree of fitting to the training set will be worse, implying that the model's bias will be greater, but this is only relative. *x* features are chosen at random for node splitting at each node of each decision tree. The lower the Gini index, the less likely it is that the selected samples in the set will be misclassified. The Gini index is expressed as follows:

(1) $$\begin{array}{c} \text{Gini}\left(s\right)=\sum_{c=1}^{C}s_{c}\left(1-s_{c}\right)\\ =1-\sum_{c=1}^{C}s_{c}^{2}, \end{array}$$

where *s*_*c*_ represents the probability that the selected sample belongs to the *c* category and then the probability that this sample is wrongly classified is (1- *s*_*c*_). The sample set has a total of *C* categories.

The RF weak classifier employs the CART number, and the CART decision tree is also known as a classification regression tree. When the dataset's dependent variable is a continuous value, the tree algorithm is a regression tree, and the mean value of leaf node observations can be used as the predicted value; when the dataset's dependent variable is a discrete value, the tree algorithm is a classification tree, which can solve the classification problem very well. However, because the algorithm is a binary tree, a nonleaf node that is a multilevel discrete variable may be used multiple times. Simultaneously, if a nonleaf node is a continuous variable, the decision tree will treat it as a discrete variable as well. The most popular feature selection methods today are information gain [20], gain rate [21], Gini coefficient [22], and the chi-square test [23].

Accuracy, precision, recall, and F1 score are commonly used evaluation indicators to assess the classification performance of the random forest algorithm. It is assumed that the number of samples classified as positive is *N*_*TP*_, and the number of samples classified as negative is *N*_*FP*_. The number of samples classified as negative that are positive is *N*_*FN*_, and the number of samples classified as negative that are negative is *N*_*TN*_. Each indicator's expression is as follows:

(2) $$\begin{array}{c} \text{Accuracy}=\frac{N_{TP}+N_{TN}}{N_{TP}+N_{FN}+N_{FP}+N_{TN}},\\ \text{Precision}=\frac{N_{TP}}{N_{TP}+N_{FP}},\\ \text{Recall}=\frac{N_{TP}}{N_{TP}+N_{FN}},\\ \text{F}1=\frac{2\times \text{Precision}\times \text{Rcall}}{\left(\text{Precision}+\text{Rcall}\right)}. \end{array}$$

**Figure 2:**
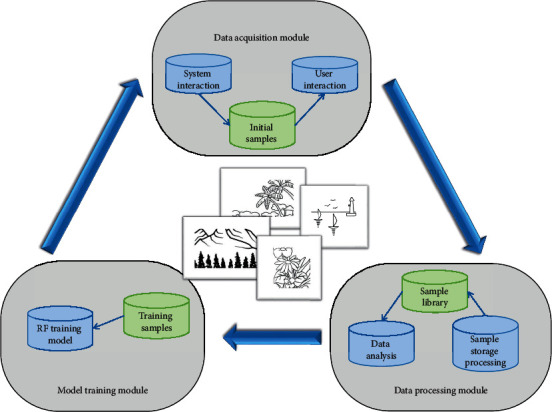
RF-based natural landscape animation self-learning framework.

The main criterion for measuring the quality of the animation after the natural landscape animation is generated is whether the user is satisfied. This article proposes an RF-based self-learning framework for natural landscape animation to improve the quality of the generated animation. The goal of self-study in natural landscape animation is to generate as much user-satisfied natural landscape animation as possible, thereby increasing the user's animation satisfaction. The framework is divided into three sections: data acquisition, data processing, and training.  Figure 2 depicts the self-learning framework for natural landscape animation proposed in this article.

The data collection module oversees gathering the user's score for scene selection. As training sample data, files containing animation information, documents containing qualitative data, and completed animations will be combined. In addition to collecting data, the data acquisition module must continuously transmit the training model's output results to the animation generation system. So that the natural landscape animation generation system can make better decisions. The following are the primary functions of the data acquisition module: first, the RF data must be gathered, which includes the animation's information frame, the candidate scenes, and the user satisfaction of a single decision tree for each candidate scene. Second, employ the voting method to determine predicted user satisfaction. Finally, compute the user recommendation score. The user recommendation score is calculated by dividing user satisfaction by the sum of all candidate scenarios' user satisfaction. Finally, the user recommendation score is incorporated into the scoring item, multiplied by the appropriate weight, and added to the overall score. The weight is determined by the number of candidate scenes; the greater the number of candidate scenes, the greater the weight.

The data processing module handles sample data storage, management, and analysis. First, the data acquisition module transmits the complete sample data, and the standard sample data are obtained after information extraction and processing, which is stored in the database, and then the module analyzes and decides the data in the sample database. The training module trains using randomly selected training samples, and the resulting learning model is returned to the data acquisition module. This module receives the data processing module's results to determine whether to continue the iterative training, ensuring that the learning model can be continuously updated with the operation of the generation system.

## 4. Experimental Results and Analysis

### 4.1. Experimental Data

The natural landscape generation system includes 88 themes, 120 template groups, and 64 scenes. Because this article employs RF, the topics and scenes are divided into 8 groups, and the templates are divided into 15 groups based on their own categories, in order to avoid too many branches of the decision tree. There are six attributes in the standard sample: theme group number, theme instance, *M*th group template instance, scene group number, and scene instance. *M* = 1, 2,…, 15. Each sample has a classification, and the classification value is one of one, two, three, or four. This classification value represents the user's score for the finished animation scene. The training sample processing flow is as follows: first, obtain the initial sample data from the generation system; then, after interacting with the user to obtain the label of a portion of the sample data; and finally, the data processing module processes the entire standard sample data as the RF training sample. In the data processing module, the self-training algorithm is used to finish labeling some unlabeled samples.

### 4.2. Experimental Parameter Settings

A total of 518 animations have been created using the natural landscape animation design method proposed in this article. The database is sampled for 300 training samples. The CART algorithm is used as the base learner in the RF model, and it is planned to create 6 base learners, so the training data for each base classifier is 50 pieces. In this article, each node of each base decision tree is selected by first selecting a subset containing *h* attributes from the node's attribute set and then selecting an optimal attribute from this subset for division. The parameter *h* = log2*s*, where *s* represents the current node's number of attributes. The effectiveness of the method proposed in this article is primarily determined by whether it increases user satisfaction. To determine whether the user's satisfaction has increased, examine whether the probability of the system selecting the user's favorite scene has increased following the self-learning system and whether the user's chances of being selected for scenes that he or she dislikes are reduced.  Table 1 depicts the experimental environment for this article.

### 4.3. Experimental Results and Analysis

Table 2 shows the details of the user's score on the satisfaction of the generated animation.

Find 12 message animations with a score of 1 from the user-scored animations, and extract the corresponding message text content and scenes. Choose 12 message animations with a score of 4, and extract the message text content and scenes, as shown in Tables 3 and 4. The contents of these 24 messages have nothing to do with one another. The 24 messages were used in the experiment to conduct a comparative test. The experiment's specific operation is as follows: (1) enter the message text into the generation system without self-learning capability, and the system will select a scene for you. Each message is tested a total of 20 times. (2) Enter the message text into the self-learning generation system to obtain the scene chosen by the system. Each message is tested a total of 20 times. (3) Compare the likelihood of disgusting/favorite scenes occurring in the two groups of experiments.

**Figure 3:**
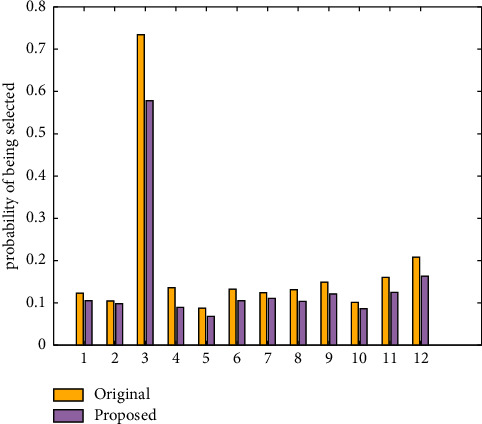
The selection probability obtained by different methods when the score is 1.

**Figure 4:**
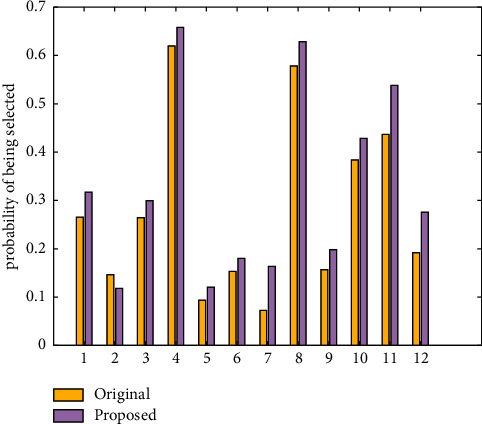
The selection probability obtained by different methods when the score is 4.

The comparison results of the selection probability of user dissatisfaction scenarios in the original animation generation system and the animation design system proposed in this article are shown in Table 5 and Figure 3.  Table 6 and Figure 4 show the comparison results of the likelihood that the user will choose the scene.

The number in the first column of Table 5 represents the scenario in which the user's name is not satisfied, a total of 12. The comparison of the data in the second and third columns of the table shows that the probability of being selected for scenes with a score of 1 using the traditional animation design method is higher than the probability of being selected using the animation design method proposed in this article. Because the animation design method proposed in this article has a self-learning function, it has the potential to increase user satisfaction. When the animation is created, it will actively avoid selecting scenes with a score of 1. As a result, the likelihood of a scene with a score of 1 being chosen is greatly reduced. Figure 3 depicts a visual comparison of the two methods based on the scene selection rate, with a score of 1. This validates the RF model introduced in this article's animation design of natural landscapes.

Table 6's first column contains 12 scene numbers that users are very happy with. The second column of data represents the likelihood that a scene with a score of 1 was chosen using the original animation design method. The third column represents the likelihood of each scene being chosen using the animation design method proposed in this article. For each row of 12 records, the data in the second column are lower than the data in the third column. It demonstrates that the method used in this article is more easily selected for user-satisfied scenarios. Favorites are more likely to be chosen, which increases user satisfaction. This is due to the self-learning function of the animation design method proposed in this article. Figure 4 depicts a visual comparison of the two methods on the scene selection rate with a score of 4. This validates the RF model introduced in this article for the design of natural landscape animation.

## 5. Conclusion

Natural landscape animation has a wide range of applications, but in a hundred people's eyes, there are a hundred Hamlets. Everyone has a different preference for animation style. This article proposes a natural landscape animation design method with self-learning function in order to propose user satisfaction. This method's central idea is to incorporate the RF model into the animation design process. RF can generate a learning model with user evaluation as the classification result to guide the automatic design of natural landscape animation, resulting in user-satisfying natural landscape animation. Simultaneously, the RF-based natural landscape animation design can continuously update the learning model based on user needs and is self-learning. The experimental results show that the animation design method proposed in this article reduces the selection rate of user dissatisfied scenes while increasing the selection rate of user-satisfied scenes. It demonstrates that the method described in this article can be used to optimize the animation design process and increase user satisfaction. However, there are some limitations to this article, such as the subjectivity of user ratings and how to improve user satisfaction further. In response to the subjectivity of user evaluations, the follow-up will use the method of increasing the number of user ratings to reduce subjectivity interference. In order to improve user satisfaction even further, this article will attempt to replace RF with other machine learning models in the future and see whether the system's performance can be further optimized.

## Figures and Tables

**Table 1:** Lab environment.

Hardware environment	CPU: Xeon E7-8890 v4 Master control server: 32G HP graphics workstation 8 render servers: ZI2I4W7-24398DMP
Software environment	The operating system is 64-bit Windows, the ontology editing tool Protégé 3.5, the database SqlServer2019, the integrated development tool Eclipse 2.0, Microsoft visual Studio2010, the animation production tool Maya2018, the rendering software BackBurner 2014

**Table 2:** Score details.

Score	Meaning
1	Dissatisfied
2	Basically satisfied
3	Quite satisfied
4	Very satisfied

**Table 3:** Messages with a score of 1.

No.	Message	Selected scene
[1]	Damaged buildings everywhere after earthquake	Damaged_building.ma
[2]	Rapid growth of weeds covering rocky ground	Weed_rock.ma
[3]	The wind blows a puff of smoke	Wind_dust.ma
[4]	Floods hit farmland	Flooded_farmland.ma
[5]	Black smoke rises from the valley	Smoky_Valley.ma
[6]	The grass is full of rubbish	Grass _litter.ma
[7]	The branches are bare and some crows stand on them	Branch_crow.ma
[8]	Thunder and lightning, a storm is coming	Lightning_strikes.ma
[9]	The strong sun shines on the ground and it hasn't rained for a long time	Sunny_ground.ma
[10]	Seeds sprouted but not grown	Seeds_germinate.ma
[11]	The grass is all withered	Withered_grass.ma
[12]	The river is polluted and aquatic life cannot survive	Polluted_river.ma

**Table 4:** Messages with a score of 4.

No.	Message	Selected scene
[13]	The grass after the rain is very fresh	Grassland.ma
[14]	The rice fields in autumn are like golden waves, and it is harvest season again	Paddy.ma
[15]	The boulder is covered with moss	Moss.ma
[16]	The waterfall rushed into the pool, making a rushing sound, and there were small fish swimming in the water	Fall.ma
[17]	pink petals falling	Petal.ma
[18]	The tops of the huge mountains are covered with snow	Mountains.ma
[19]	The sun shines on the ice, it's starting to thaw	Ice.ma
[20]	There are insects chirping in the grass by the lake in the summer night	Grass_ lake.ma
[21]	The aspen trees in the two rows of the road grow neatly	Poplar.ma
[22]	White clouds floated in the sky, and a flock of birds flew by	Cloud_bird.ma
[23]	Various colored berries in the grass	Berry.ma
[24]	People sitting around a campfire in the woods in winter	Bonfire.ma

**Table 5:** Experimental results with a score of 1.

No.	Traditional animation design method	The animation design method proposed in this article
[1]	0.1232	0.1053
[2]	0.1051	0.0983
[3]	0.7346	0.5785
[4]	0.1165	0.0896
[5]	0.0876	0.0682
[6]	0.1328	0.1055
[7]	0.1244	0.1107
[8]	0.1314	0.1039
[9]	0.1490	0.1212
[10]	0.1011	0.0865
[11]	0.1602	0.1248
[12]	0.2081	0.1637

**Table 6:** Experimental results with a score of 4.

No.	Traditional animation design method	The animation design method proposed in this article
[13]	0.2657	0.3173
[14]	0.1468	0.1183
[15]	0.2643	0.2995
[16]	0.6198	0.6582
[17]	0.0941	0.1205
[18]	0.1532	0.1804
[19]	0.0725	0.1639
[20]	0.5787	0.6285
[21]	0.1570	0.1982
[22]	0.3842	0.4287
[23]	0.4369	0.5382
[24]	0.1923	0.2760

## Data Availability

The labeled dataset used to support the findings of this study is available from the corresponding author upon request.
